# Npro of Classical Swine Fever Virus Suppresses Type III Interferon Production by Inhibiting IRF1 Expression and Its Nuclear Translocation

**DOI:** 10.3390/v11110998

**Published:** 2019-10-31

**Authors:** Tong Cao, Xiaoye Li, Yonghao Xu, Shengnan Zhang, Zuohuan Wang, Ying Shan, Jianhe Sun, Weihuan Fang, Xiaoliang Li

**Affiliations:** 1 Institute of Preventive Veterinary Medicine, Zhejiang Provincial Key Laboratory of Preventive Veterinary Medicine, Zhejiang University, Hangzhou 310058, China; 2 Experimental Animal Center of Zhejiang Academy of Medical Sciences, Hangzhou 310013, China; 3 Shanghai Key Laboratory of Veterinary Biotechnology, Shanghai 200240, China

**Keywords:** classical swine fever virus, Type III interferons, Npro, IRF1

## Abstract

Classical swine fever virus (CSFV) causes a contagious disease of pigs. The virus can break the mucosal barrier to establish its infection. Type III interferons (IFN-λs) play a crucial role in maintaining the antiviral state in epithelial cells. Limited information is available on whether or how CSFV modulates IFN-λs production. We found that IFN-λ3 showed dose-dependent suppression of CSFV replication in IPEC-J2 cells. Npro-deleted CSFV mutant (∆Npro) induced significantly higher IFN-λs transcription from 24 h post-infection (hpi) than its parental strain (wtCSFV). The strain wtCSFV strongly inhibited IFN-λs transcription and IFN-λ3 promoter activity in poly(I:C)-stimulated IPEC-J2 cells, whereas ∆Npro did not show such inhibition. Npro overexpression caused significant reduction of IFN-λs transcription and IFN-λ3 promoter activity. Both wtCSFV and ∆Npro infection induced time-dependent IRF1 expression in IPEC-J2 cells, with ΔNpro showing more significant induction, particularly at 24 hpi. However, infection with wtCSFV or Npro overexpression led not only to significant reduction of IRF1 expression and its promoter activity in poly(I:C)-treated IPEC-J2 cells but also to blockage of IRF1 nuclear translocation. This study provides clear evidence that CSFV Npro suppresses IRF1-mediated type III IFNs production by inhibiting IRF1 expression and its nuclear translocation.

## 1. Introduction

Innate immune activation upon viral infection is dependent on recognition by pattern recognition receptors (PRRs). PRRs, such as Toll-like receptors (TLRs), the cytosolic retinoic acid-inducible gene I (RIG-I) and melanoma differentiation-associated protein 5 (MDA-5), recognize viral RNA as PAMPs (pathogen associated molecular patterns) and respond by activating signaling networks that culminate in the induction of interferons (IFNs) and establish an antiviral state, which helps to limit viral infection [1,2]. Type I IFNs (IFN-α and IFN-β) were initially considered as the major components of the host innate antiviral responses [3]. Recently, type III IFNs (IFN-λs) have been demonstrated as new members of antiviral cytokines. Type III IFNs comprise four members in humans: IFN-λ1, IFN-λ2, IFN-λ3 and IFN-λ4 [4,5,6,7]. For swine, IFN-λ1, IFN-λ3 and IFN-λ4 have been reported, while IFN-λ2 is probably deficient [8,9].

The signaling networks and expression processes between types I and III IFNs are similar [10,11]. However, there are still many differences between the two types of IFNs. Production of type I IFNs requires all IFN enhanceosome components, while type III IFNs can be induced by independent regulation of interferon regulatory factors (IRF) or NF-κB [12]. Another main difference is that receptors for type I IFNs are ubiquitous, while receptors for type III IFNs are largely confined to mucosal epithelia [13]. Therefore, type III IFNs play critical roles in forming the first line of antiviral defense in the respiratory and gastrointestinal tracts. While IRFs and NF-κB are essential regulators for type III IFNs, IRF1 may play a particular role in this process [14]. The mitochondrial antiviral signaling protein (MAVS) is a central player in the antiviral signaling cascade and localizes to mitochondria and peroxisomes. Mitochondrial MAVS is responsible for induction of type I IFNs, whereas peroxisomal MAVS activates type III IFNs-dependent signal pathways [15,16]. Peroxisomal MAVS in intestinal epithelial cells is critical for IRF1-mediated type III IFNs activation [14]. Intestinal epithelial cells express a low level of type I IFNs receptors and respond poorly to type I IFNs [17]. However, they produce type III IFNs in abundance upon porcine epidemic diarrhea virus infection [9]. The robust response of type III IFNs to invading viruses is due to differentiation of epithelial cells and up-regulation of peroxisomes biogenesis [14].

Classical swine fever (CSF) is a highly contagious disease caused by classical swine fever virus (CSFV) and leads to important economic losses to the pig industry [18]. Infection with CSFV may lead to hemorrhages and petechiae on the lymphoid organs, kidneys and urinary bladder, and necrosis and ulceration of the small intestine, colon, and ileocecal valve [19]. CSFV is a single-stranded, positive sense RNA virus with an approximate genome size of 12,300 nt. The genome contains a single open reading frame (ORF) flanked by a 5’-untranslated region (UTR) and a 3’-UTR, and encodes a polyprotein of 3898 amino acids. The polyprotein is processed by cellular and viral proteases to yield four structural proteins and eight non-structural proteins [20]. CSFV, together with bovine viral diarrhea virus (BVDV) and border disease virus, constitutes the *Pestivirus* genus in the *Flaviviridae* family [21]. A unique feature of the pestiviruses compared with the other genera of the family *Flaviviridae* is presence of the Npro gene at the 5’ end of the single large ORF [22,23,24]. CSFV subverts innate immune defenses by preventing type I IFNs induction, a property mediated by the viral protein Npro independent of other viral elements [25,26,27,28]. Npro of CSFV interferes with type I IFNs synthesis by inducing proteasomal degradation of IRF3, thus allowing the virus to establish a productive infection in host cells [29,30].

Unlike the well-documented interactions between CSFV and type I IFNs, interaction between CSFV and type III IFNs remains largely unknown. A recent study showed that type III IFNs could be detected in the supernatant of BVDV-infected bovine plasmacytoid dendritic cells (pDCs) or in serum from BVDV-infected animals [31]. Infection with CSFV was able to induce limited expression of type III IFNs in PK-15 cells and in animal tissues [32]. However, no information is available regarding whether and how CSFV modulates type III IFNs response and what roles IRF1 might play in this process. In the present study, we show that CSFV infection strongly suppressed type III IFNs production in the poly(I:C) stimulated cells, and such suppression was mainly due to Npro protein. We further demonstrate that IRF1 is a positive regulator of type III IFNs and CSFV Npro down-regulates type III IFNs by suppressing IRF1 expression and its nuclear translocation. Our findings expand the current understanding of CSFV in deploying its Npro to escape from host innate antiviral mechanisms.

## 2. Materials and Methods

### 2.1. Virus, Cells and Recombinant Plasmids

The CSFV Shimen strain (wtCSFV) is maintained in our laboratory and used for construction of the Shimen strain-based cDNA clone (pA-Shimen). PK-15 cells (porcine kidney cells) were cultured at 37 °C and 5% CO_2_ in Dulbecco’s minimal essential medium (DMEM, Thermo Fisher Scientific, USA) supplemented with 5% fetal bovine serum (FBS, Hyclone, Logan, UT, USA), 100 U/mL penicillin, and 100 µg/mL streptomycin (Thermo Fisher Scientific, New York, NY, USA). IPEC-J2 cells (porcine intestinal epithelial cells) were cultured at 37 °C and 5% CO_2_ in minimal essential medium (MEM F12, Thermo Fisher Scientific) supplemented with 10% FBS (Hyclone), 100 U/mL penicillin, and 100 µg/mL streptomycin (Thermo Fisher Scientific).

The pCMV-flag vector (Beyotime, Shanghai, China) was used for construction of the eukaryotic expression vectors. To obtain the recombinant vector pCMV-Npro-flag, the CSFV Npro gene was amplified from pA-Shimen and cloned into the pCMV-flag vector fused to the N-terminal of the flag tag. Similarly, the recombinant vector pCMV-IRF1-flag was constructed by amplifying the porcine IRF1 gene (GenBank No. NM_001097413.1) from cDNA of the IPEC-J2 cells.

### 2.2. Virus Rescue and Identification

The mutant CSFV Shimen strain with Npro deletion (∆Npro) was constructed from pA-Shimen. The fragment without the Npro gene of pA-Shimen was amplified with the primer pair ∆Npro-F/∆Npro-R (Table S1). The resulting amplicon contained 15-bp overlaps at both ends. The two ends were joined together by the Gibson assembly method (Vazyme, China), resulting in the new cDNA clone pA-∆Npro. The plasmid was transformed into *E. coli* DH10B competent cells. Bacterial clones containing the correct deletion were identified by sequencing. The genome RNA of ∆Npro was transcribed in vitro and electroporated into PK-15 cells according to the previous study [33]. To rescue the mutant virus, continuous passaging proceeded by subculturing the electroporated cells (or previous passage cells) into new T-25 flasks (in 1:3 ratio) every 2–3 days. A small portion of each passage was seeded in a 24-well plate to detect CSFV E2 protein expression by indirect immunofluorescence assay (IFA) as described below. The passaging ended when significant amount of fluorescence was observed. The cultures of each passage were subjected to cycles of freezing and thawing. The supernatant samples were harvested and the rescued virus was identified for the Npro gene deletion by reverse-transcription PCR (using the primer pair outside of the Npro gene) and sequencing. Stocks of the rescued virus were stored at −80 °C.

### 2.3. Single Step Growth Curve

Growth kinetics of the virus ∆Npro was compared to its parental wtCSFV. Monolayer IPEC-J2 cells were infected with wtCSFV or ∆Npro (MOI = 0.1) in 24-well plates and adsorbed for 1 h in 5% CO_2_ at 37 °C. The supernatant was then removed and 600 μL of fresh media was added. Samples of the whole cultures (both supernatant and cells) were collected at 6, 12, 18, 24, 30, 36, 42 and 48 h post-infection (hpi) for viral titration (TCID_50_).

### 2.4. Virus Titration and Indirect Immunofluorescence

The rescued virus was titrated using the end-point dilution method. PK-15 cells were seeded onto 96-well plates (10^4^ cells in 100 μL medium per well) and inoculated with 10-fold serial dilutions of the viral suspensions. Cells were grown for 72 h at 37 °C and 5% CO_2_, and fixed with 80% ice-cold acetone at −20 °C for 30 min. The anti-E2 monoclonal antibody 6B8 [34] was diluted (1:5000) in PBS containing 0.5% skimmed milk powder, and incubated for 1 h at 37 °C. The wells were washed three times with PBS and incubated with Alexa-Fluor-labeled anti-mouse secondary antibody (488 nm; Thermo Fisher Scientific) at 37 °C for 1 h. The cells were washed and examined under a fluorescence microscope (X81, Olympus, Japan). Titers were calculated according to the method of Reed and Münch and presented as median tissue culture infective dose (TCID_50_)/mL.

### 2.5. Virus Infection, Treatments with IFN-λ3 and Chemicals, and Transfection

For examination of the antiviral activity of type III IFNs against CSFV, IPEC-J2 cells were treated with IFN-λ3 (Bio-Techne, Minneapolis, MN, USA) for 12 h and inoculated with wtCSFV or ∆Npro strains. Cells were then washed and replenished with fresh medium containing IFN-λ3. The whole cultures were collected at 12, 24, 36, and 48 hpi, and viral titers (TCID_50_) were examined for each collection as the index of antiviral activity of IFN-λ3. For dose-response analysis, IPEC-J2 cells were pretreated with 10, 100, or 1000 ng/mL of IFN-λ3 for 12 h and infected with wtCSFV or ∆Npro for 24 h in the presence of IFN-λ3 and virus titers were examined. The anti-CSFV activity of IFN-λ3 (100 ng/mL) in IPEC-J2 cells were further examined 12 h before, at the time of, or after inoculation with wtCSFV or ∆Npro, and the whole cultures were collected at 24 hpi for virus titration.

In order to examine modulation of transcription of IFN-λs or IRF1 by CSFV or Npro protein, IPEC-J2 cells were inoculated with wtCSFV or ∆Npro strain (MOI = 1) or transfected with the plasmid pCMV-Npro for 24 h, and then stimulated with 100 ng/mL poly(I:C) (Invivogen, USA) for 12 h. The cells were then lysed at various time points post-infection to analyze the transcription levels of IFN-λs or IRF1 by qRT-PCR using the primers listed in Table S2.

To examine regulation of type III IFNs by IRF1, over-expression and RNA silencing of IRF1 were used. IPEC-J2 cells were transfected with pCMV-IRF1 for 24 h and then lysed to analyze the transcription levels of the IFN-λs by qRT-PCR. For gene silencing, IPEC-J2 cells were transfected with the small interfering RNA (siRNA) against IRF1 (Table S3) for 24 h with scrambled control siRNA (siNC) included as a control (Genepharma, Shanghai, China). Delivery of poly(I:C), plasmids or siRNAs into IPEC-J2 cells was conducted by using Lipofectamine 2000 (Thermo Fisher Scientific) according to the manufacturer’s instructions. The cells were then stimulated with 100 ng/mL poly(I:C) for 12 h. The cells were lysed for transcriptional analysis of IFN-λs by qRT-PCR. Expression of the target proteins was confirmed by Western blotting.

### 2.6. RNA Isolation and qRT-PCR

IPEC-J2 cells treated as described above were washed once with Hanks’ balanced salt solution (Beyotime, China) and lysed directly in cell culture dishes. Total RNA was isolated with the RNA isolation kit according to the manufacturer’s instructions (Bioteke, Beijing, China) and transcribed to cDNA by using HiScript III 1st Strand cDNA Synthesis Kit (+gDNA wiper) (Vazyme, Nanjing, China). The synthesized cDNA was subjected to quantitative PCR using SYBR green PCR mix according to the manufacturer’s instructions (Vazyme) with the Stratagene Mx3005P real-time PCR system (Agilent Technologies, Santa Clara, CA, USA). The β-actin gene was used as an internal control for each run. Specificity was confirmed by sequencing of the PCR products and melting-curve analysis for qPCR. The threshold cycle (C_T_) values for target genes (*IFN-λ1*, *IFN-λ3*, *IFN-λ4* and *IRF1*) and the differences in their C_T_ values (ΔC_T_) were determined. Relative transcription levels of the target genes were presented as fold changes relative to the respective controls by using the 2^-ΔΔC^_T_ method.

### 2.7. Dual Luciferase Reporter Assay

To examine the activity of the IFN-λ3 or IRF1 promoter during CSFV infection, IPEC-J2 cells were grown in 24-well plates to about 80% confluence and transfected with the luciferase reporters (pGL3-P_IFN-λ3_ or pGL3-P_IRF1_) and pRL-TK (Promega, USA) at a ratio of 10:1 for 12 h [35]. Cells were then infected with wtCSFV or ∆Npro mutant at MOI of 1 for 24 h, followed by stimulation with poly(I:C) for 12 h. For confirmation of Npro as a viral antagonist against IFN-λ3 or IRF1, IPEC-J2 cells were inoculated into 24-well plates and transfected with pCMV-Npro, the luciferase reporter (pGL3-P_IFN-λ3_ or pGL3-P_IRF1_) and pRL-TK at a ratio of 10:10:1 for 24 h. Cells were then stimulated with 10 ng/mL of poly(I:C) for 12 h. For examination of the IFN-λ3 signaling regulated by IRF1, IPEC-J2 cells were transfected with pCMV-IRF1, the luciferase reporter (pGL3-P_IFN-λ3_) and pRL-TK for 24 h at a ratio of 10:10:1. Or, IPEC-J2 cells were transfected with luciferase reporter pGL3-P_IFN-λ3_, siIRF1 plasmid or siNC plasmid, and pRL-TK for 24 h at a ratio of 10:10:1, and then stimulated with poly(I:C) for 12 h. The promoter sequences of IFN-λ3 and IRF1 were obtained at http://www-bimas.cit.nih.gov/molbio/proscan/. Transfection was performed by using Lipofectamine 2000 according to the manufacturer’s instructions. The dual luciferase assay was conducted according to the previous study [35]. Promoter activity was expressed as the ratio of enzyme activity values (light units) of firefly luciferase to *Renilla* luciferase. Relative promoter activity in the test samples was normalized to mock-infected/transfected cells.

### 2.8. Western Blotting

Western blotting was performed to detect changes of the expression of target molecules in cells infected with wtCSFV or ∆Npro mutant, or cells transfected with plasmids or siRNAs. Briefly, cells at the indicated time points were harvested and protein concentration in the samples was quantified using a bicinchoninic acid assay kit (MultiSciences, Shanghai, China). The proteins were separated by 12% SDS-PAGE and electro-transferred onto PVDF membranes (Millipore, Billerica, MA, USA). The blots were blocked for 1 h with 5% skimmed milk and then incubated overnight at 4 °C with the following primary antibodies: mouse anti-E2 monoclonal antibody (produced in our laboratory), rabbit anti-Npro polyclonal antibody (produced in our laboratory), mouse anti-β-actin monoclonal antibody (MultiSciences, China), rabbit anti-IRF1 monoclonal antibody (Abcam, Shanghai, China), or mouse anti-flag monoclonal antibody (Abclonal, China). The blots were then washed and incubated for 1 h at 37 °C with goat anti-rabbit or goat anti-mouse antibodies conjugated to horseradish peroxidase (HRP) (KPL, Gaithersburg, MD, USA). The blots were visualized by using SuperSignal West Pico chemiluminescent substrate (Thermo Fisher Scientific, USA) and images were captured in a Gel 3100 chemiluminescent imaging system (Sagecreation, Beijing, China). The grey density of protein bands on the blots was analyzed with the Gel-Pro analyzer software and abundance of IRF1 in various treatments was expressed relative to that under mock conditions after normalization with β-actin.

### 2.9. Confocal Microscopic Analysis of IRF1 Nuclear Translocation Affected by CSFV

Confocal microscopy was conducted to examine possible regulation of CSFV or the Npro protein on nuclear translocation of IRF1. IPEC-J2 cells were cultured in Petri dishes (10 mm in diameter, Cellvis, USA) and inoculated with wtCSFV or ∆Npro mutant at MOI of 1. After infection for 24 h at 37 °C and 5% CO_2_, the infected cells were stimulated with 100 ng/mL poly(I:C) for 12 h. For transfection of the pCMV-Npro-flag vector, IPEC-J2 cells were seeded into Petri dishes and grown to about 80% confluence. pCMV-Npro-flag was then delivered into cells with Lipofectamine 2000 for 12 h, followed by replacement with fresh medium. The cells were incubated for 12 h to allow gene expression and then transfected with 100 ng/mL poly(I:C) for another 12 h. Confocal microscopic analysis was conducted according to the previous study [36] with the following primary antibodies: rabbit anti-IRF1, mouse anti-E2 or anti-flag monoclonal antibody.

### 2.10. Statistical Analysis

All data were expressed as mean ± SD of three independent experiments. Statistical analyses were conducted by using GraphPad Prism 6 for analysis of variance (ANOVA). Asterisks *, ** or *** in figures indicate statistical significance at the *P* < 0.05, *P* < 0.01 or *P* < 0.001 level, respectively.

## 3. Results

### 3.1. Identification and Growth Kinetics of the ∆Npro Mutant

**Figure 1:**
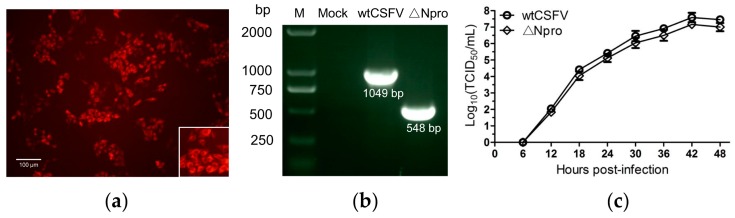
Identification of the Npro deletion mutant of CSFV (ΔNpro) and its growth in IPEC-J2 cells. The supernatant of the third passage culture of the cells electroporated with ΔNpro RNA was inoculated into PK-15 cells for E2 protein detection by IFA (**a**). Deletion of the Npro gene was identified by reverse transcription PCR using primer pairs outside of the target gene with the wtCSFV included for comparison (**b**). Growth kinetics of ΔNpro in comparison with wtCSFV by virus titers (TCID_50_) (**c**).

To examine the role of Npro in type III IFNs response, the Npro gene-deleted mutant was constructed from its cDNA clone. Rescuing of the ∆Npro mutant in PK-15 cells was identified by IFA and sequencing. Only sparse fluorescent cells could be detected 72 h after electroporation. However, the number of fluorescent cells increased significantly in the following passages. Infection of PK-15 cells with the culture supernatant samples collected at the third passage led to generation of ∆Npro progeny viruses (Figure 1a). RT-PCR analysis of the viral genomic RNA showed that the ∆Npro mutant virus was 501 bp shorter than its parental strain (Figure 1b), indicating absence of the Npro gene. 

To determine if Npro deletion would alter viral replication, growth kinetics of the ∆Npro mutant was evaluated and compared with its parental wtCSFV strain in IPEC-J2 cells. Figure 1c shows that ∆Npro had slightly lower replication than its parental strain, but with no significant difference. These data suggest that Npro deletion does not have a significant impact on viral replication.

### 3.2. Antiviral Activities of IFN-λ3 against CSFV

**Figure 2:**
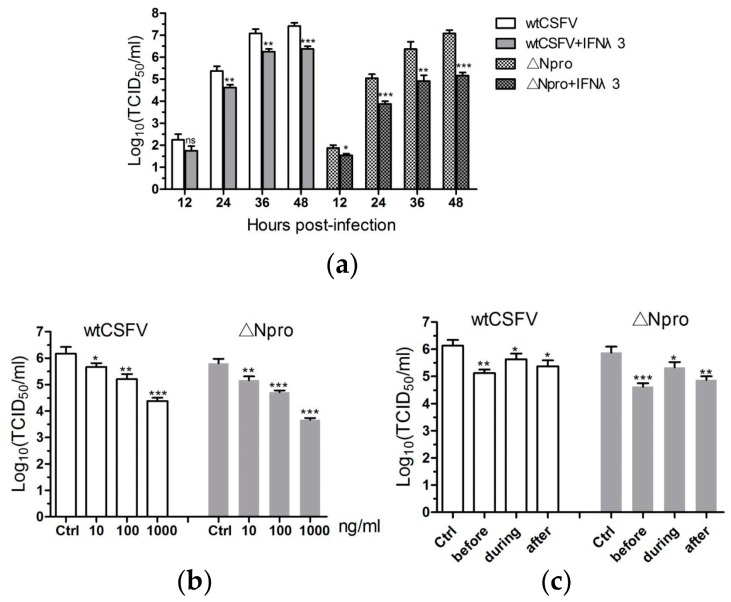
IFN-λ3 exerts antiviral activity against CSFV in IPEC-J2 cells. Suppression of wtCSFV and ΔNpro replication by IFN-λ3 was time-dependent (**a**) and dose-dependent (**b**), and differed with timing of treatment **(c**). Results were presented as mean ± SD of three independent experiments: *, *P* < 0.05; **, *P* < 0.01; ***, *P* < 0.001.

Previous studies have demonstrated that type I IFNs could inhibit replication of pestiviruses [37,38,39], so we deduced that type III IFNs might have similar antiviral activity against CSFV. Figure 2a shows that the viral titers of wtCSFV and ∆Npro were significantly reduced by IFN-λ3 treatment, particularly from 24 to 48 hpi. IFN-λ3 inhibited wtCSFV and ∆Npro in a dose-dependent manner: the higher the concentration of IFN-λ3, the stronger the inhibition of viral replication with 1.5 to over 2 log decrease of the virus titers at 1000 ng/mL treatment (Figure 2b). Figure 2c indicates that IFN-λ3 inhibited CSFV replication when applied before, during or after infection, while the highest level of inhibition was seen when applied before infection. These data demonstrate that IFN-λ3 possessed anti-CSFV activity.

### 3.3. Npro Deletion Mutant Induced Much Higher Levels of Type III IFNs Transcription than Its Parental CSFV Strain

**Figure 3:**
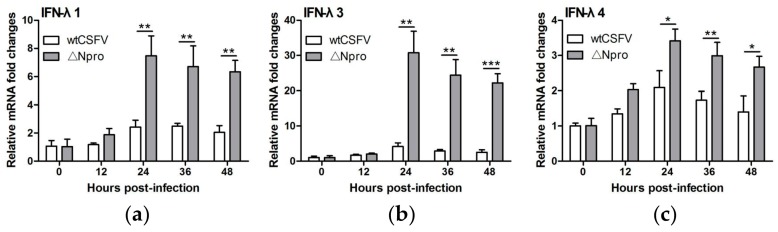
Expression of type III IFNs in IPEC-J2 cells infected with wtCSFV and ΔNpro by quantitative reverse transcription PCR. IFN-λ1 (**a**), IFN-λ3 (**b**), and IFN-λ4 (**c**). Results were presented as mean ± SD of three independent experiments: *, *P* < 0.05; **, *P* < 0.01, ***, *P* < 0.001.

Two PAMPs can be sensed by cellular receptors during CSFV infection: one is the viral genomic ssRNA and the other double-strand RNA (dsRNA), an intermediate replication product of the virus. TLR-7 binds with the ssRNA derived from CSFV and TLR-3 senses the replication intermediate dsRNAs [40,41]. We expected that CSFV infection would induce production of type III IFNs in IPEC-J2 cells as a result of signaling via the TLRs. However, wtCSFV infection only resulted in a limited increase of transcription of IFN-λs starting from 24 hpi, while infection with ∆Npro significantly up-regulated the transcription of IFN-λs that peaked at 24 hpi and was maintained at higher levels at 36 and 48 hpi (*P* < 0.05, 0.01 or 0.001 in comparison with wtCSFV, Figure 3). These findings suggest that Npro of CSFV might be involved in down-regulating expression of type III IFNs. 

### 3.4. CSFV Inhibited poly(I:C)-Induced Production of Type III IFNs by Its Npro

**Figure 4:**
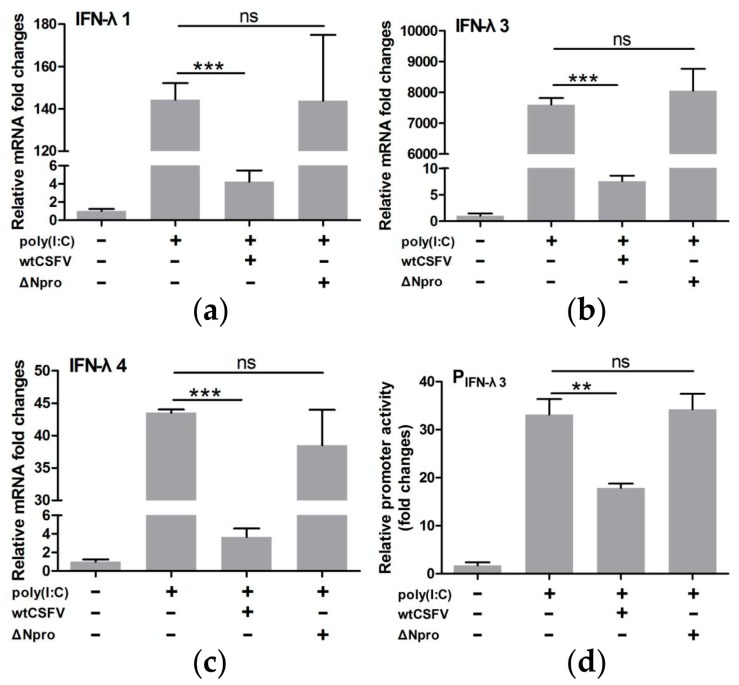
Inhibition of type III IFNs production in poly(I:C)-stimulated IPEC-J2 cells by wtCSFV but not by its mutant ΔNpro. Quantification of IFN-λ1 (**a**), IFN-λ3 (**b**), and IFN-λ4 (**c**) by reverse transcription PCR. IFN-λ3 promoter activity as determined by luciferase reporter assay (**d**). Results were presented as mean ± SD of three independent experiments: **, *P* < 0.01; ***, *P* < 0.001.

Because wtCSFV infection induced a lower level of expression of IFN-λs than the ∆Npro mutant (Figure 3), we hypothesized that CSFV might be involved in inhibiting the host type III IFNs response by Npro protein as observed in type I IFNs [25,26,27,28]. To investigate this possibility, qRT-PCR was performed on virus-infected and poly(I:C)-stimulated cells. Results shows that infection with wtCSFV significantly down-regulated transcription of IFN-λs in poly(I:C) treated cells. However, Npro deletion reversed expression of IFN-λs to levels similar to poly(I:C)-treated and mock-infected cells (*P* > 0.05) (Figure 4a–c). Since IFN-λ3 was more sensitive to poly(I:C) stimulation, its promoter was selected for dual-luciferase reporter assay to further confirm the role of CSFV Npro in suppression of IFN-λs in IPEC-J2 cells. Figure 4d reveals that wtCSFV infection significantly down-regulated poly(I:C)-induced IFN-λ3 promoter activity (*P* < 0.01, compared with mock-infected cells). Deletion of Npro reverted the promoter activity similar to poly(I:C)-treated control cells (*P* > 0.05). These data suggest that CSFV infection suppresses expression of IFN-λs probably by its Npro protein.

**Figure 5:**
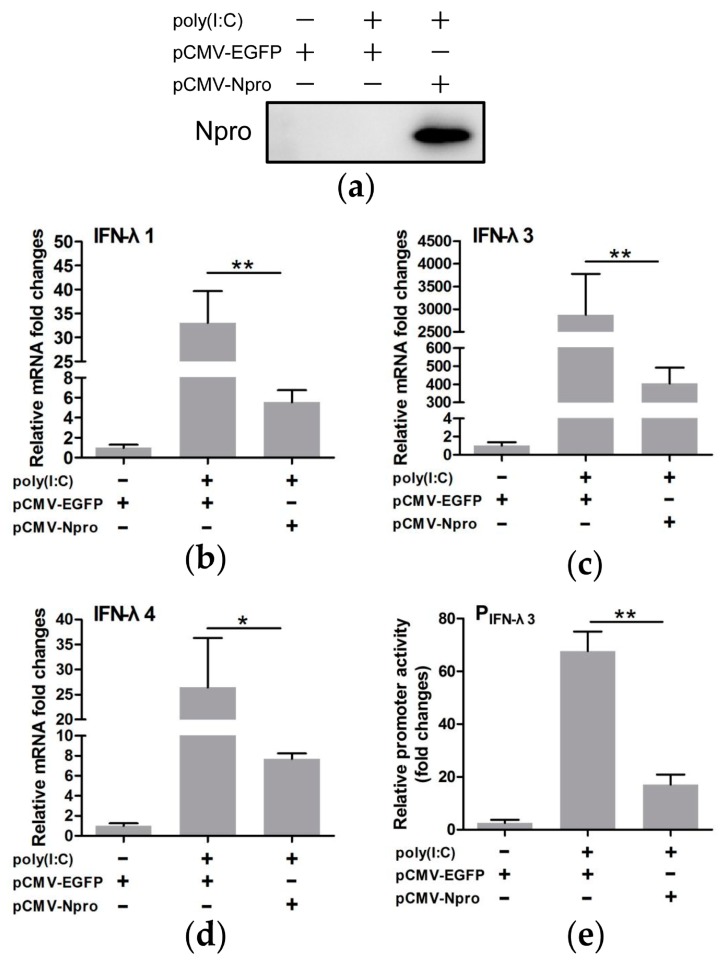
CSFV Npro contributed to inhibition of type III IFNs in IPEC-J2 cells. Npro protein expression was evaluated by Western blotting (**a**). CSFV Npro expression in IPEC-J2 cells transfected with pCMV-Npro inhibited mRNA transcription of IFN-λ1 (**b**), IFN-λ3 (**c**) and IFN-λ4 (**d**) shown as fold changes of mRNA after normalization using β-actin mRNA. IFN-λ3 promoter activity was suppressed by CSFV Npro expression (**e**). Recombinant plasmid expressing EGFP (pCMV-EGFP) was used as a control. Results were presented as mean ± SD of three independent experiments: *, *P* < 0.05; **, *P* < 0.01.

To further identify the role of Npro as the viral antagonist of type III IFNs, Npro protein was overexpressed to examine its effect on transcription of type III IFNs and IFN-λ3 promoter activity. Western blotting showed that the Npro gene was expressed efficiently (Figure 5a). Npro expression significantly down-regulated transcription of IFN-λs and inhibited the IFN-λ3 promoter activity in poly(I:C)-treated cells (*P* < 0.05 or 0.01, as compared with pCMV-EGFP control vector) (Figure 5b–e). These data lead to the conclusion that Npro is a potent viral antagonist to expression of type III IFNs in CSFV infected IPEC-J2 cells. 

### 3.5. IRF1 Was a Positive Regulator of Type III IFNs

**Figure 6:**
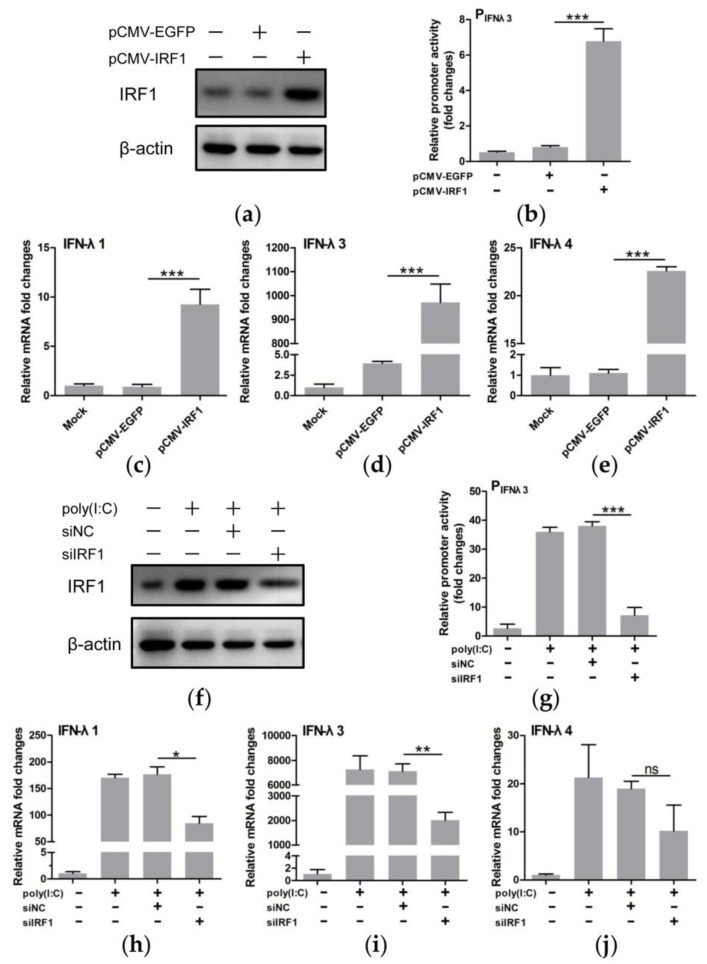
IRF1 is a positive regulator of type III IFNs in IPEC-J2 cells. Overexpression of IRF1 in IPEC-J2 cells transfected with pCMV-IRF1 or pCMV-EGFP (control) was verified by Western blotting using anti-IRF1 antibody (**a**). Npro expression increased IFN-λ3 promoter activity (**b**). Induction of IFN-λ1 (**c**), IFN-λ3 (**d**) and IFN-λ4 (**e**) by overexpression of IRF1 in IPEC-J2 cells, shown as fold changes of mRNA after normalization using β-actin mRNA. Silencing of IRF1 (siIRF1) in the poly(I:C) stimulated IPEC-J2 cells using small interfering RNA assessed by Western blotting (**f**). Knock-down of IRF1 expression inhibited IFN-λ3 promoter activity (**g**), and down-regulated mRNA transcription of IFN-λ1 (**h**), IFN-λ3 (**i**) and IFN-λ4 (**j**) shown as fold changes of mRNA after normalization using β-actin mRNA. Recombinant plasmid expressing EGFP (pCMV-EGFP) or scrambled RNA (siNC) was used as a control. Results were presented as mean±SD of three independent experiments: *, *P <* 0.05; **, *P* < 0.01; ***, *P* < 0.001.

A recent study showed that IRF1 knockdown down-regulated transcription of IFN-λ1 in Huh7 cells, suggesting the role of IRF1 in regulation of IFN-λ1 [14]. To further investigate if IRF1 regulates expression of IFN-λs, we overexpressed IRF1 by delivering the pCMV-IRF1 vector into IPEC-J2 cells (Figure 6a). IRF1 overexpression led to significant elevation of the IFN-λ3 promoter activity and increased transcription of IFN-λs (*P* < 0.001) (Figure 6b–e). We also attempted to examine the effect of IRF1 down-regulation by small RNA interference (Figure 6f) on transcription of IFN-λs in IPEC-J2 cells stimulated with poly(I:C). IRF1 silencing decreased IFN-λ3 promoter activity (*P* < 0.001) (Figure 6g) and reduced the transcription of IFN-λs in poly(I:C)-treated cells with IFN-λ1 and IFN-λ3 having statistical significance (*P* < 0.05 or 0.01) (Figure 6h–j). These results demonstrate the role of IRF1 in positive regulation of type III IFNs in IPEC-J2 cells.

### 3.6. IRF1 Expression Profiles Were Different between wtCSFV and ΔNpro Infected Cells

**Figure 7:**
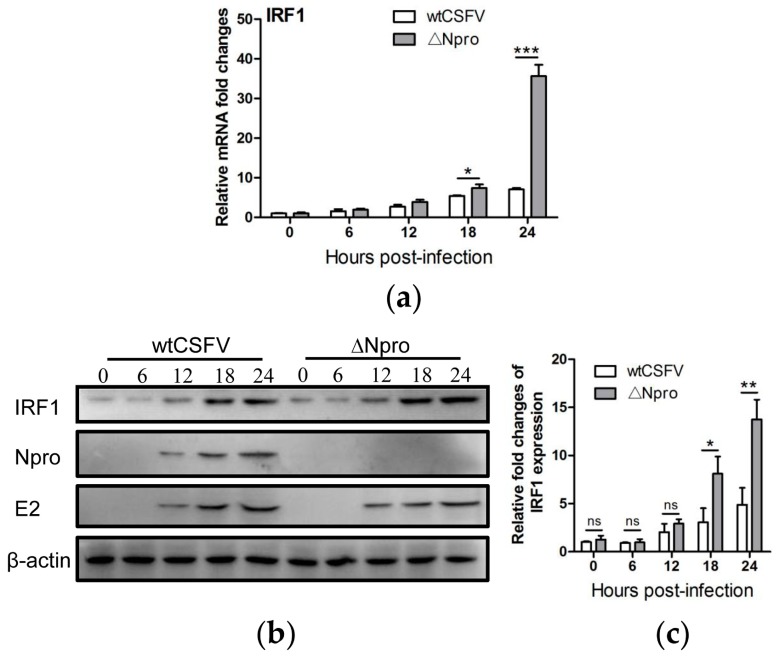
Expression of IRF1 in IPEC-J2 cells infected with wtCSFV and ΔNpro. Fold changes of IRF1 expression in IPEC-J2 cells during infection with wtCSFV or its ΔNpro mutant: relative IRF1 mRNA transcription (**a**), IRF1 expression by Western blotting (**b**) and semi-quantitative analysis of IRF1 expression shown as the density ratio of IRF1 to β-actin relative to mock infection (**c**). Results were presented as mean ± SD of three independent experiments: *, *P* < 0.05; **, *P* < 0.01; ***, *P* < 0.001.

In IPEC-J2 cells not subjected to poly(I:C) stimulation, CSFV infection induced time-dependent increase of IRF1 expression at the mRNA and protein levels (Figure 7a–c). However, the Npro deletion mutant induced higher expression of IRF1 than its parental strain particularly at 24 hpi. This is similar to the expression profiles of IFN-λs between ∆Npro and wtCSFV (Figure 3), suggesting that Npro might down-regulate expression of both IFN-λs and IRF1.

### 3.7. Down-Regulation of IRF1 Expression by CSFV Npro

**Figure 8:**
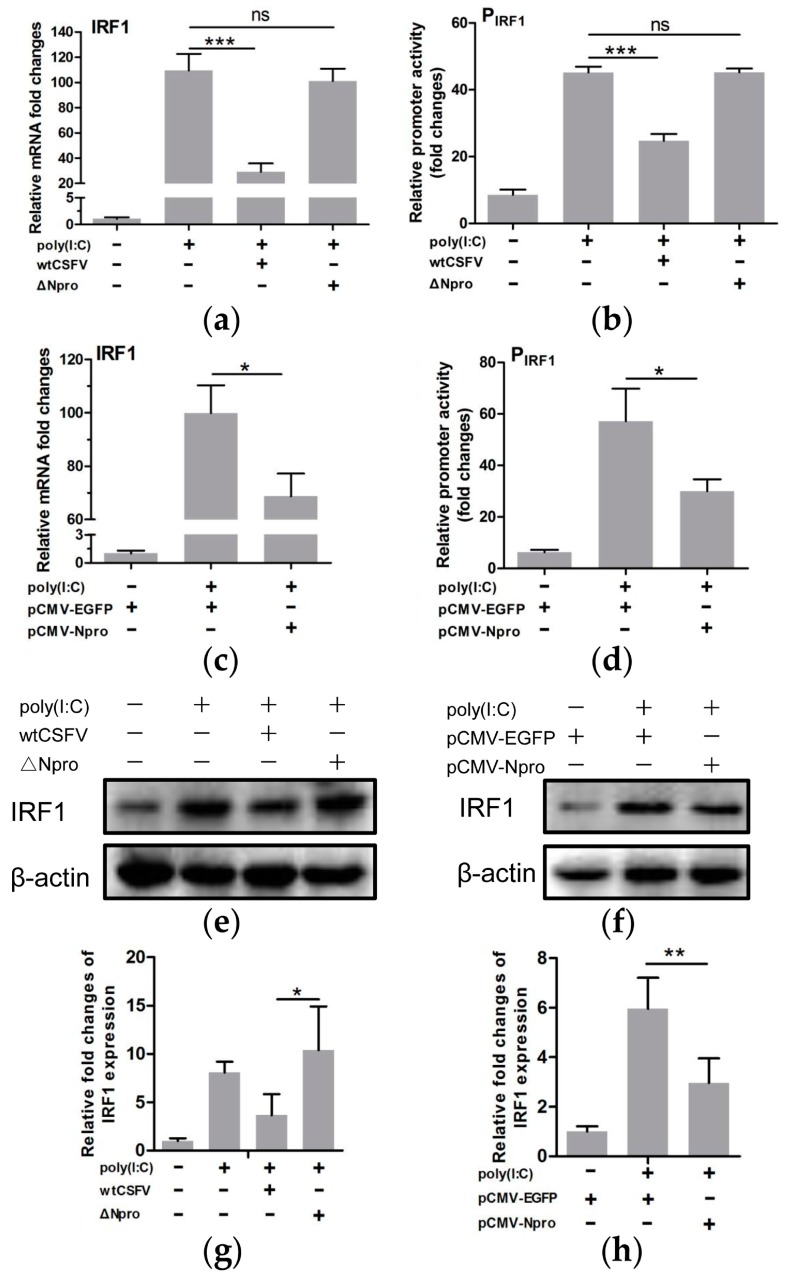
Npro of CSFV inhibited poly(I:C)-stimulated expression of IRF1 in IPEC-J2 cells. IRF1 transcription (**a**) and IRF1 promoter activity (**b**) of IPEC-J2 cells infected with wtCSFV and ΔNpro in response to poly(I:C) stimulation. IRF1 transcription (**c**) and IRF1 promoter activity (**d**) of IPEC-J2 cells transfected with pCMV-Npro or control vector pCMV-EGFP in response to poly(I:C) stimulation. Western blot analysis of IRF1 protein expression in wtCSFV- or ∆Npro-infected (**e**) or in Npro-overexpressed (**f**) cells. Relative IRF1 expression was shown as the density ratio of IRF1 to β-actin relative to mock treatment (**g** and **h**). Results were presented as mean ± SD of three independent experiments: *, *P* < 0.05; **, *P* < 0.01; ***, *P* < 0.001.

As IRF1 positively regulated the transcription of IFN-λs, and the promoter activities of the IFN-λs were significantly down-regulated upon CSFV infection, we hypothesized that CSFV might be involved in regulating type III IFNs by affecting IRF1 expression. We found a significant reduction of IRF1 mRNA in poly(I:C)-stimulated cells infected by wtCSFV in comparison with uninfected but poly(I:C)-treated cells (Figure 8a, *P* < 0.001). Infection by the ∆Npro mutant did not affect poly(I:C)-induced IRF1 mRNA level (*P* > 0.05). The luciferase reporter assay also showed significant suppression of the IRF1 promoter activity by wtCSFV infection (*P* < 0.001), but not by ∆Npro (*P* > 0.05) (Figure 8b). To further identify if Npro was directly involved in suppression of IRF1, the recombinant plasmid pCMV-Npro was transfected into IPEC-J2 cells. Figure 8c indicates that Npro overexpression did decrease IRF1 transcription (*P* < 0.05, in comparison with control cells expressing EGFP). The IFN-λ3 promoter activity was also reduced in the Npro-overexpressed cells (Figure 8d). Western blotting showed that either infection with wtCSFV or Npro overexpression led to decreased expression of the IRF1 protein in poly(I:C) stimulated cells while the ∆Npro mutant strain did not have inhibitory effect (Figure 8e–h). The above data indicate that Npro exhibited negative regulation of IRF1 expression.

### 3.8. Inhibition of IRF1 Nuclear Translocation by CSFV Npro

**Figure 9:**
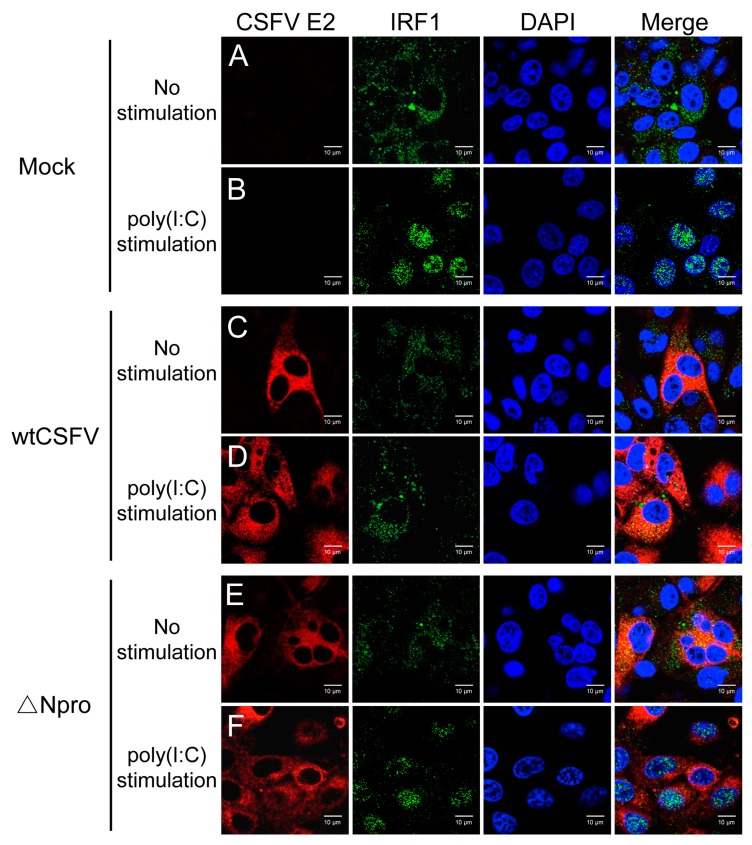
Npro of CSFV inhibited nuclear translocation of IRF1 in IPEC-J2 cells. Identification of IRF1 nuclear translocation in mock-infected IPEC-J2 cells (panel **A** and **B**), or cells infected with wtCSFV (panel **C** and **D**) or ΔNpro (panel **E** and **F**) in response to poly(I:C) stimulation for IFA. Cells were fixed and stained with anti-IRF1 antibody and anti-E2 antibody followed by probing with Alexa Fluor 555-conjugated goat anti-rabbit antibody and Alexa Fluor 488-conjugated goat anti-mouse secondary antibody to visualize IRF1 and E2, respectively. Nuclei were stained with DAPI. The experiments were repeated three times, and images of one typical experiment were shown.

IRF1 contains a functional nuclear localization signal (NLS) located right after the DNA-binding domain. IRF1 is diffusely distributed in the cytoplasm, but could be instantly activated upon stimulation and translocates to the nucleus [42]. To examine whether CSFV blocked IRF1 nuclear translocation, IPEC-J2 cells were infected with wtCSFV or ∆Npro for 24 h and stimulated with poly(I:C) for confocal imaging of IRF1 distribution. IRF1 was present in the cytoplasm of unstimulated cells (Figure 9, panel A). Stimulation by poly(I:C) promoted translocation of IRF1 to the nuclei (Figure 9, panel B). Infection with wtCSFV inhibited IRF1 nuclear translocation induced by poly(I:C) stimulation while ∆Npro did not inhibit such translocation (Figure 9, panel D and F), indicating that Npro might be involved in blocking IRF1 nuclear translocation.

**Figure 10:**
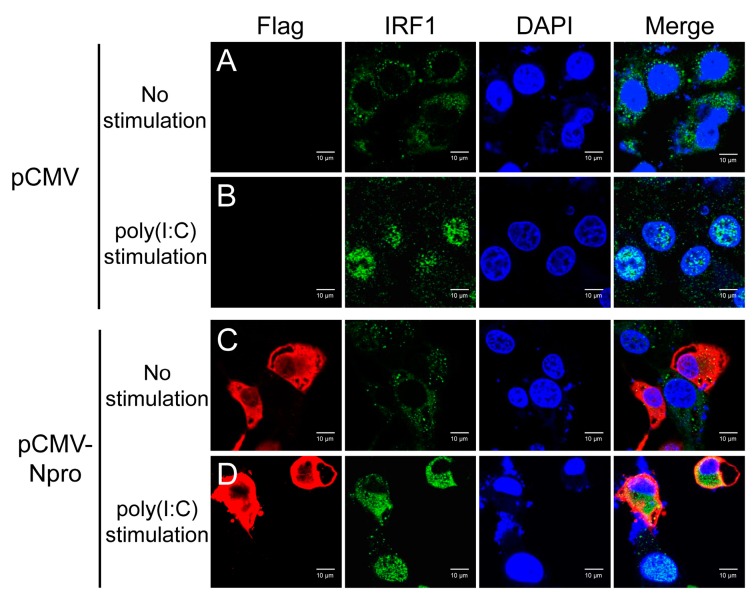
Overexpression of Npro inhibited nuclear translocation of IRF1 in IPEC-J2 cells. Identification of IRF1 nuclear translocation in IPEC-J2 cells transfected with pCMV (as control, panel **A** and **B**) or pCMV-Npro-flag (panel **C** and **D**) in response to poly(I:C) stimulation for IFA. Cells were fixed and stained with anti-IRF1 antibody and anti-flag antibody followed by probing with Alexa Fluor 555-conjugated goat anti-rabbit antibody and Alexa Fluor 488-conjugated goat anti-mouse secondary antibody to visualize IRF1 and Npro, respectively. Nuclei were stained with DAPI. The experiments were repeated three times, and images of one typical experiment were shown.

IRF1 was typically diffused in the cytoplasm after transfection with the control pCMV vector, and translocated into the nucleus upon poly(I:C) stimulation (Figure 10, panel A and B). When Npro-expressing cells were stimulated by poly(I:C), IRF1 was still shown in the cytoplasm (Figure 10, panel D). These data demonstrate that CSFV Npro blocked nuclear translocation of IRF1.

## 4. Discussion

Understanding mechanisms of viral strategy to evade from host antiviral responses is critical for elucidation of the pathogenesis of CSFV infection. Type I IFNs represent one of the first lines of defense of the innate immune system and are produced rapidly upon recognition of viral factors by PRRs. CSFV is known to interfere with the generation of type I IFNs by inducing proteasomal degradation of IRF3 [29,43,44]. Type III IFNs play a critical role in antiviral innate immunity in mucosal epithelial cells [45,46,47]. However, little information is available as to whether and how CSFV modulates type III IFN response. The present study demonstrates that the Npro protein of CSFV inhibited IRF1-mediated type III IFNs production by down-regulation of IRF1 expression and blockage of its nuclear translocation.

The Npro-deleted mutant showed similar growth kinetics to its parental strain, indicating that CSFV Npro does not alter the replication in cell culture, which is consistent with the previous study [48]. Some viruses have evolved to encode viral antagonists for evasion of type III IFNs responses [49,50,51]. Reid et al showed the induction of type III IFNs in pDCs upon BVDV infection, and type III IFNs contributed to suppression of cellular immune response during BVDV infection [31]. Cai et al shows that CSFV induced the expression of type III IFNs in PK-15 cells, but with a limited level of increase, suggesting that CSFV might have a strategy to suppress expression of type III IFNs [32]. We also found limited up-regulation of type III IFNs transcription upon wtCSFV infection in IPEC-J2 cells, though the transcription level peaked at 24 hpi and decreased thereafter, which is different from the results shown by Cai et al that the mRNA level of IFN-λ1 and IFN-λ3 induced by wtCSFV peaked at 36 and 48 hpi, respectively. We speculate that the difference might be due to different cell types used. Because CSFV causes enteric pathologies, a cell line from the intestines could be a good choice. The IPEC-J2 cell line was from non-transformed cells collected from the jejunum of a neonatal piglet and suitable for infection studies for enteropathogens [52]. IPEC-J2 exhibited robust expression of type III IFNs with a relatively lower type I IFNs response [9]. Thus, the IPEC-J2 cell line was chosen in this study. Cell lines or primary cultures from other intestinal areas or from adult pigs could be approached for further investigation of the interactions between CSFV and type III IFNs.

We found that the ∆Npro mutant induced significantly higher transcription of IFNs in IPEC-J2 cells from 24 to 48 hpi than its parental strain, suggesting that the Npro protein might be antagonistic to expression of type III IFNs. Because CSFV Npro has been found to subvert the type I IFN response [25,26,27,28], we speculated if Npro might have similar effect on type III IFNs. We showed that wtCSFV strongly inhibited expression of poly(I:C)-induced type III IFNs in IPEC-J2 cells and Npro deletion abolished such an inhibitory effect. We further showed that Npro over-expression led to a marked reduction of mRNA transcripts of type III IFNs. Either infection with wtCSFV or over-expression of Npro also resulted in significant reduction of the IFN-λ3 promoter activity of poly(I:C)-treated IPEC-J2 cells. These results demonstrate that CSFV employs its Npro to inhibit type III IFNs expression probably by targeting the regulatory elements involved in transcription of IFN-λs.

Activation of type III IFNs, as the case with type I IFNs, requires IRF3, IRF7 and NF-κB [11]. However, expression of type III IFNs does not need all components of the enhanceosome [12]. IRF1 is a predominant antiviral molecule against many viruses [53]. It is the first identified member of the IRF family, but it does not induce production of type I IFNs [54]. Odendall et al has shown that IRF1 regulates peroxisome-mediated IFN-λ1 expression [14]. Epithelial cells have preference of producing IFN-λs over IFN-α/β upon viral infection [55,56]. This may be due to relatively higher expression of peroxisomes in epithelial cells [15]. Peroxisomal MAVS is known to induce type III IFNs, as opposed to induction of type I IFNs via mitochondrial MAVS [14]. To better understand the regulation of type III IFNs by IRF1, we attempted to regulate levels of IRF1 expression by overexpression or RNA interference in IPEC-J2 cells. Overexpression of IRF1 led to a significant increase of transcription of all IFN-λs and IFN-λ3 promoter activity, while gene silencing showed opposite effects, indicating that IRF1 positively regulates expression of type III IFNs.

We observed that CSFV infection induced limited up-regulation of IRF1 expression in IPEC-J2 cells. This is similar to the finding reported by Li et al that CSFV infection induced IRF1 expression in PK-15 cells [57]. However, such induction is more pronounced with the Npro-deleted mutant than the wtCSFV strain. Significant suppression of IRF1 expression or its promoter activity was also seen in wtCSFV infected or Npro-expressing cells stimulated by poly(I:C). These findings indicate that Npro is the antagonistic viral component to IRF1 expression. It is known that there is a binding site upstream of IRF1 for p65, suggesting that NF-κB is a positive regulator of IRF1 expression [58]. Chen et al. reported that CSFV infection failed to induce p65 nuclear translocation [59]. It might be that CSFV Npro down-regulates IRF1 by antagonizing the NF-κB activity because CSFV Npro is known to interact with the NF-κB inhibitor IκBα [60]. However, the exact mechanisms of Npro-IκBα interaction in affecting p65 nuclear translocation await further investigation. Besides, some TLRs have been demonstrated to be involved in modulating type III IFNs production [61,62,63]. However, Cao et al showed that TLR3-mediated immune responses induced by poly(I:C) were inhibited upon CSFV Shimen strain infection [64]. Therefore, modulating the sensor molecules in TLR signaling pathways may also be a strategy for CSFV to establish efficient infection.

IRF1 is mostly distributed in the cytoplasm of unstimulated cells. When activated, IRF1 is phosphorylated and translocates to the nucleus, thus regulating the production of IFNs [42]. In order to escape from the immune surveillance, viruses have evolved strategies to interfere with nuclear translocation of IRF1 [9,65]. We wondered if CSFV could block nuclear translocation of IRF1. We found that infection with the wtCSFV strain or overexpression of Npro inhibited nuclear translocation of IRF1 in poly(I:C)-stimulated cells, while the ∆Npro mutant did not affect such translocation. These results demonstrate that CSFV deploys Npro to prevent IRF1 from entering into the nuclei. 

In conclusion, this study clearly shows that CSFV suppresses production of type III IFNs in swine intestinal epithelial cells via inhibition of IRF1 expression and nuclear translocation, and that its Npro protein contributes to such suppressive effects. Our findings provide insights into the mechanisms of CSFV pathogenicity in breaching the host intestinal epithelial cell defense by down-regulating type III IFNs expression. We speculate that there might be similar mechanisms in the airway epithelial cells where CSFV gains its entry into the host via aerosol infection. Further studies are required to examine if Npro inhibits IRF1 and type III IFNs expression in other epithelial cells including airway epithelial cells and how Npro inhibits IRF1 expression and blocks its nuclear translocation.

## Supplementary Materials

The following are available online at https://www.mdpi.com/1999-4915/11/11/998/s1, Table S1: Primers for construction of the Npro-deleted cDNA clone, Table S2: Real-time PCR primers for IFN-λs and IRF1, Table S3: Small interfering RNAs (siRNAs) for IRF1.
