# Supplementary material for: Npro of Classical Swine Fever Virus Suppresses Type III Interferon Production by Inhibiting IRF1 Expression and Its Nuclear Translocation
Source: Viruses. 2019 Oct 31;11(11):998. doi: 10.3390/v11110998 (PMC6893713; doi:10.3390/v11110998)
Supplement: Supplementary file 1 [file viruses-11-00998-s001.pdf]

Table S1. Primers for construction of the Npro-deleted cDNA clone

Gene product	primers	Sequences (5′-3′)
pA-ΔNpro	ΔNpro-F	TGCTGTACATGGCACATGTCTGATGATGGCGC AAGTGGAAGTAAAG
	ΔNpro-R	GTGCCATGTACAGCAGAGATTTTTATACTAG

The overlap sequence is underlined.

Table S2. Real-time PCR primers for IFN-λs and IRF1

Gene		Primer sequences (5′-3′)
IFN-λ1	sense	ATGGCTACAGCTTGGATCGTGGTG
	antisense	GAGGGGAGAGCTGCAGCTCC
IFN-λ3	sense	CCTTCAAGAGGGCCAAGGATGCC
	antisense	GTGAAGGGGCTGGTCCAGGC
IFN-λ4	sense	GTCCGTGTCTGCCTTGTCACTGAC
	antisense	AGGACTTTGGGCCCTTTCACTATGC
IRF1	sense	GCAGGACTTGGACATTGAACAGGCC
	antisense	CTCAGTTAATTTCCCCTCCTCGTCCTCATC
β-actin	sense	CCAGCACGATGAAGATCAAGATC
	antisense	CAAATGCTTCTAGGCGGACTG

Table S3. Small interfering RNAs (siRNAs) for IRF1

Target gene		Sequences (5′-3′)
IRF1	sense	GGAAAGAGAGAAAGUCCAAGUU
	antisense	AACUUGGACUUUCUCUCUUUCC
control	sense	UUCUCCGAACGUGUCACGU
	antisense	ACGUGACACGUUCGGAGAA
